# Supplementary figures and images for: Critical appraisal of minimally invasive keyhole surgery for intracranial meningioma in a large case series
Source: PLoS One. 2022 Jul 28;17(7):e0264053. doi: 10.1371/journal.pone.0264053 (PMC9333232; doi:10.1371/journal.pone.0264053)

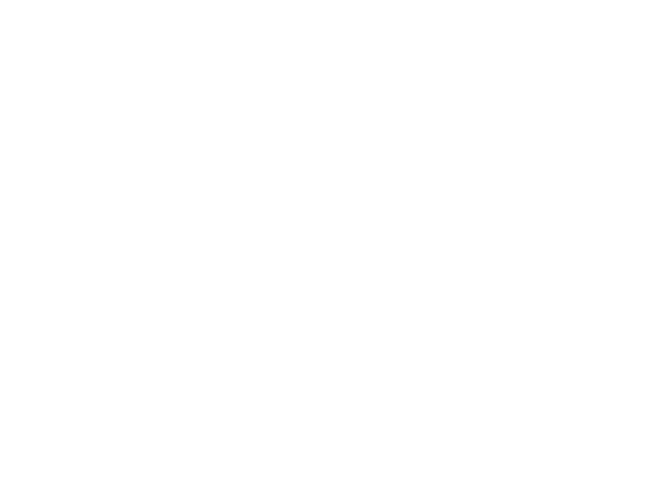

Supplement: S1 Fig — The corresponding GIF animation of the 6 approaches illustrated in Fig 1. (GIF) [file pone.0264053.s001.gif]
