# Supplementary material for: Critical appraisal of minimally invasive keyhole surgery for intracranial meningioma in a large case series
Source: PLoS One. 2022 Jul 28;17(7):e0264053. doi: 10.1371/journal.pone.0264053 (PMC9333232; doi:10.1371/journal.pone.0264053)
Supplement: S1 Table — A breakdown of number of cases in which keyhole and traditional approaches were used for skull base and non-skull base meningiomas. (DOCX) [file pone.0264053.s002.docx]

**Supplemental Table 1: Keyhole and Traditional Approaches Used for Skull Base and Non-Skull Base Meningiomas**

| **Tumor descriptive** |  |
| --- | --- |
| **Location** | **Total number of Operations (%)** |
|  |  |
| **Skull Base Meningiomas** | 240 (66%) |
| **Non-Skull Base Meningiomas** | 126 (34%) |
|  |  |
| **Type of Approach** |  |
|  |  |
| ***Keyhole Approaches*** | **213** |
| Endoscopic Endonasal | 74 (35%) |
| Supraorbital | 73 (34%) |
| Retromastoid | 38 (18%) |
| Mini-pterional | 20 (9%) |
| Suboccipital | 4 (2%) |
| Transfalcine | 4 (2%) |
|  |  |
| ***Traditional Approaches*** | **153** |
| Convexity/Parafalcine | 104 (68%) |
| Pterional Skull Base | 17 (11%) |
| Open Middle Fossa | 13 (9%) |
| Suboccipital Foramen Magnum | 6 (4%) |
| Pterional non-skull base | 5 (3%) |
| Bifrontal Skull base | 4 (2%) |
| Tentorium non-skull base | 3 (2%) |
| Intraventricular | 1 (1%) |
|  |  |
| **Total** | **366** |
