# Supplementary material for: Critical appraisal of minimally invasive keyhole surgery for intracranial meningioma in a large case series
Source: PLoS One. 2022 Jul 28;17(7):e0264053. doi: 10.1371/journal.pone.0264053 (PMC9333232; doi:10.1371/journal.pone.0264053)
Supplement: S2 Table — Preoperative factors limiting GTR and factors predicting progression are analyzed using multivariate binomial and cox regression analysis. (DOCX) [file pone.0264053.s003.docx]

**Supplemental Table 2: Multivariate Regression Analysis for Tumor Resection and Progression**

| **Binomial Multivariate Regression for Predicting Preoperative Factors Limiting GTR** | | | | |
| --- | --- | --- | --- | --- |
|  |  |  |  |  |
| **Categorical** | **p value** | **RR** | **95% C.I.** | |
|  |  |  | **Lower** | **Upper** |
| Prior Radiation | 0.10 | 9.3 | 0.65 | 133 |
| Prior Surgery | 0.08 | 5.0 | 0.80 | 31.1 |
| Invasion to CS-MC-Orbit-ITF/ Adherence to neurovascular structures | **<0.001** | 30.4 | 12.3 | 75.1 |
|  |  |  |  |  |
|  |  |  |  |  |
| **Binomial Multivariate Cox Regression for Predicting Progression** | | |  |  |
|  |  |  |  |  |
| **Categorical** | **p value** | **Odds Ratio** | **95% C.I.)** | |
|  |  |  | Lower | Upper |
| Prior surgery or XRT | 0.06 | 0.45 | 0.19 | 1.0 |
| Adherence to neurovascular structures | 0.20 | 0.48 | 0.16 | 1.5 |
| Invasion to CS-MC-Orbit-ITF | 0.53 | 0.74 | 0.29 | 1.9 |
| GTR/NTR | 0.54 | 0.72 | 0.24 | 2.1 |
|  |  |  |  |  |
