# Supplementary material for: Critical appraisal of minimally invasive keyhole surgery for intracranial meningioma in a large case series
Source: PLoS One. 2022 Jul 28;17(7):e0264053. doi: 10.1371/journal.pone.0264053 (PMC9333232; doi:10.1371/journal.pone.0264053)
Supplement: S3 Table — A breakdown of the cranial nerve outcomes and recovery for patients undergoing keyhole meningioma surgery. (DOCX) [file pone.0264053.s004.docx]

**Supplemental Table 3: Cranial Nerve Outcomes in 193 Patients Undergoing Keyhole Meningioma Removal**

|  | **I** | **II** | **III** | **IV** | **V** | **VI** | **VII** | **VIII** | **IX** | **X** | **XI** | **XII** |
| --- | --- | --- | --- | --- | --- | --- | --- | --- | --- | --- | --- | --- |
| **Pre-OP CN Deficit** | 4 | 54 | 19 | 6 | 25 | 18 | 1 | 13 | 2 | 0 | 0 | 0 |
|  |  |  |  |  |  |  |  |  |  |  |  |  |
| **Recovery** |  |  |  |  |  |  |  |  |  |  |  |  |
| 90-day postop Worsening CND | 0 | 0 | 0 | 0 | 0 | 0 | 0 | 0 | 0 | 0 | 0 | 0 |
| Worsening from SRS/Tumor Progression | 0 | 3 (5.6%) | 1 (5.2%) | 0 | 0 | 1 (5.6%) | 0 | 2 (15.3%) | 0 |  |  |  |
| Stable | 2 (50%) | 20 (37%) | 9 (47.4%) | 3 (50%) | 3 (12%) | 7 (38.9%) | 1 (100) | 6 (46.2%) | 0 | 0 | 0 | 0 |
| Partial Rec | 1 (25%) | 19 (35.2%) | 4 (21.1%) | 3 (50%) | 13 (52%) | 8 (44.4%) | 0 | 4 (30.8%) | 0 | 0 | 0 | 0 |
| Complete Rec | 1 (25%) | 12 (22.2%) | 5 (26.3%) | 0 | 9 (36%) | 2 (11.1%) | 0 | 1 (7.7%) | 2 (100%) | 0 | 0 | 0 |
|  |  |  |  |  |  |  |  |  |  |  |  |  |
| **New CN Deficit** | 2 | 2 | 1 | 0 | 1 | 1 | 1 | 0 | 0 | 0 | 0 | 0 |
|  |  |  |  |  |  |  |  |  |  |  |  |  |
| **Recovery** |  |  |  |  |  |  |  |  |  |  |  |  |
| Worsen | 1 (50%) | 0 | 1(100%) | 0 | 0 | 0 | 0 | 0 | 0 | 0 | 0 | 0 |
| Stable | 1 (50%) | 2 (100%) | 0 | 0 | 0 | 1 (100) | 0 | 0 | 0 | 0 | 0 | 0 |
| Partial Recovery | 0 | 0 | 0 | 0 | 0 | 0 | 1 (100%) | 0 | 0 | 0 | 0 | 0 |
| Complete  Recovery | 0 | 0 | 0 | 0 | 0 | 0 | 0 | 0 | 0 | 0 | 0 | 0 |
