# Supplementary material for: Critical appraisal of minimally invasive keyhole surgery for intracranial meningioma in a large case series
Source: PLoS One. 2022 Jul 28;17(7):e0264053. doi: 10.1371/journal.pone.0264053 (PMC9333232; doi:10.1371/journal.pone.0264053)
Supplement: S1 Video — Illustrative case examples of 3 anterior cranial fossa meningiomas: 1) tuberculum sella meningioma approached via endoscopic endonasal route, 2) olfactory groove meningioma approached via supraorbital route; 3) clinoidal meningioma approached via supraorbital route. (DOCX) [file pone.0264053.s005.docx]

<https://drive.google.com/file/d/115jlSuP6QkFrpoBw-PpV_3zrYm2Ycphb/view?usp=sharing>
