# Supplementary material for: Critical appraisal of minimally invasive keyhole surgery for intracranial meningioma in a large case series
Source: PLoS One. 2022 Jul 28;17(7):e0264053. doi: 10.1371/journal.pone.0264053 (PMC9333232; doi:10.1371/journal.pone.0264053)
Supplement: S2 Video — Illustrative case examples of 3 meningiomas: 1) petroclival meningioma approached via retromastoid route, 2) tentorial meningioma approached via suboccipital sitting position route; 3) falx meningioma approached via transfalcine gravity-assisted route. (DOCX) [file pone.0264053.s006.docx]

<https://drive.google.com/file/d/112fL0mV5GcqLxwYBYlHisSMN1Vymb7ln/view?usp=sharing>
